# Potential of Biofermentative Unsulfated Chondroitin and Hyaluronic Acid in Dermal Repair

**DOI:** 10.3390/ijms23031686

**Published:** 2022-02-01

**Authors:** Antonella D’Agostino, Annalisa La Gatta, Antonietta Stellavato, Donatella Cimini, Luisana Corsuto, Marcella Cammarota, Maria D’Agostino, Chiara Schiraldi

**Affiliations:** 1 Department of Experimental Medicine, Section of Biotechnology, Medical Histology and Molecular Biology, University of Campania Luigi Vanvitelli, 80138 Naples, Italy; annalisa.lagatta@unicampania.it (A.L.G.); antonietta.stellavato@unicampania.it (A.S.); luisanacorsuto@hotmail.it (L.C.); marcella.cammarota@unicampania.it (M.C.); maria92md@libero.it (M.D.); 2 Department of Environmental, Biological and Pharmaceutical Sciences and Technologies, University of Campania L. Vanvitelli, 81100 Caserta, Italy; donatella.cimini@unicampania.it

**Keywords:** chondroitin, skin model, hyaluronan gels, wound healing, ECM biomarkers, *striae distensae*

## Abstract

Chondroitin obtained through biotechnological processes (BC) shares similarities with both chondroitin sulfate (CS), due to the dimeric repetitive unit, and hyaluronic acid (HA), as it is unsulfated. In the framework of this experimental research, formulations containing BC with an average molecular size of about 35 KDa and high molecular weight HA (HHA) were characterized with respect to their rheological behavior, stability to enzymatic hydrolysis and they were evaluated in different skin damage models. The rheological characterization of the HHA/BC formulation revealed a G’ of 92 ± 3 Pa and a G″ of 116 ± 5 Pa and supported an easy injectability even at a concentration of 40 mg/mL. HA/BC preserved the HHA fraction better than HHA alone. BTH was active on BC alone only at high concentration. Assays on scratched keratinocytes (HaCaT) monolayers showed that all the glycosaminoglycan formulations accelerated cell migration, with HA/BC fastening healing 2-fold compared to the control. In addition, in 2D HaCaT cultures, as well as in a 3D skin tissue model HHA/BC efficiently modulated mRNA and protein levels of different types of collagens and elastin remarking a functional tissue physiology. Finally, immortalized human fibroblasts were challenged with TNF-α to obtain an in vitro model of inflammation. Upon HHA/BC addition, secreted IL-6 level was lower and efficient ECM biosynthesis was re-established. Finally, co-cultures of HaCaT and melanocytes were established, showing the ability of HHA/BC to modulate melanin release, suggesting a possible effect of this specific formulation on the reduction of stretch marks. Overall, besides demonstrating the safety of BC, the present study highlights the potential beneficial effect of HHA/BC formulation in different damage dermal models.

## 1. Introduction

Skin integrity and texture of the dermal matrix are correlated to the restoration of the biomacromolecules that contribute to preserve hydration and assembly of the extracellular matrix (ECM), and to the biochemical determinants able to counteract oxidative stress and more generally aging-related damages. The ECM of dermal tissue includes diverse fibrous molecules (collagen, elastin, fibrillin, fibronectin and laminin) and glycosamminoglycans, such as hyaluronic acid (HA), dermatan sulfate (CS-B) and chondroitin sulfate (CS-A,C) that play a key role in providing structural support, mechanical elasticity, hydration and many other properties [1]. The beneficial effects of HA are well known and widely exploited in aesthetic medicine [2,3,4], whereas CS has received attention mainly as a medical device in the orthopedic and ophthalmic fields [2,5,6]. Recent research papers reported on the use of CS in scaffolds also containing gelatin or collagen, eventually crosslinked or stabilized, for wound healing applications and as dermal substitutes [7]. CS and, generally, sulphated GAGs were shown to promote the initial cellular attachment of human dermal fibroblasts to 2D scaffolds and induce keratinocytes differentiation in keratinocyte-hMSC co-culture models, by modulating various epithelial markers [8]. In our research unsulfated chondroitin were used and obtained by fermentation of the recombinant *E. coli* K4 strain EcK4r3, and purified by ultrafiltration/diafiltration, alcohol precipitation and active carbon treatments [9]. This product is here addressed as biofermentative unsulfated chondroitin (BC), a CS variant, since it consists of the same repeating disaccharide unit of CS, formed by D-glucuronic acid and N-acetyl-D-galactosamine, but, like HA, without any sulfate groups. Recent studies have already demonstrated the beneficial effects and the biological activity of this BC on chondrocytes, compared to extractive CS. In particular, BC promoted cell proliferation and concurrently preserved the chondrocyte phenotype. It also showed improved anti-inflammatory properties compared to CS, reducing cytokines levels in a primary human chondrocyte inflammation model [10]. Previous studies, combining hyaluronic acid at higher and lower molecular weight (HCC), were investigated for dermal application and showed beneficial effects in tissue damage [11]. The use of hyaluronic acid at molecular weight ranging from 30–300 prompt cell migration and often modulate pro-inflammatory cytokines production [12]. The HCC showed prolonged stability in vitro [11] and in vivo [13]. Finally, the filling ability and the hydration capacity of injective formulation of linear HA are known to be related to HA molecular weight and concentration.

In addition, semi-synthetic CS obtained through biotechnological process and with different sulfation patterns were compared to extractive CS on a wound healing model, and in relation to the binding to TGF-beta [14]. However, the effect of unsulfated BC in dermal models has never been studied or reported in scientific literature. Therefore, in this research work we attempted to establish diverse in-vitro models’ representative for dermal damage to evaluate the effect of BC alone and in combination with HA on specific biomarkers. The combination with HA was aiming to improve the viscoelastic behavior in addition the investigation was exploring also potentially occurring synergic biochemical effect. Specifically, the present study aimed (1) to characterize the rheology and stability of HA/BC gels and hence their suitability for topic applications or injective purposes, and (2) to compare the biological response solicited by BC and HHA/BC gels with respect to dermal repair and skin rejuvenation. Specifically, HA/BC complexes were obtained exploiting the NAHYCO™ technology, already used to obtain stabilized gels based on cooperative hydrogen bonding between high and low molecular weight HA chains. Specifically, the biochemical and biological characterization was focused on exploring the effect on the dermal wound closure rate by time lapse experiments. The expression of the main biomarkers involved in matrix remodeling was studied comparing the different products in 2D cultures and also in a 3D skin model. In addition, dermal cells were challenged for inflammation with TNFα to compare the potential anti-inflammatory properties of the formulations. Finally, co-cultures of HaCaT and melanocytes investigated a further potential application of HHA/BC, in particular for the treatment of *striae distansae* [15].

## 2. Results

### 2.1. Rheological Measurements

**Figure 1:**
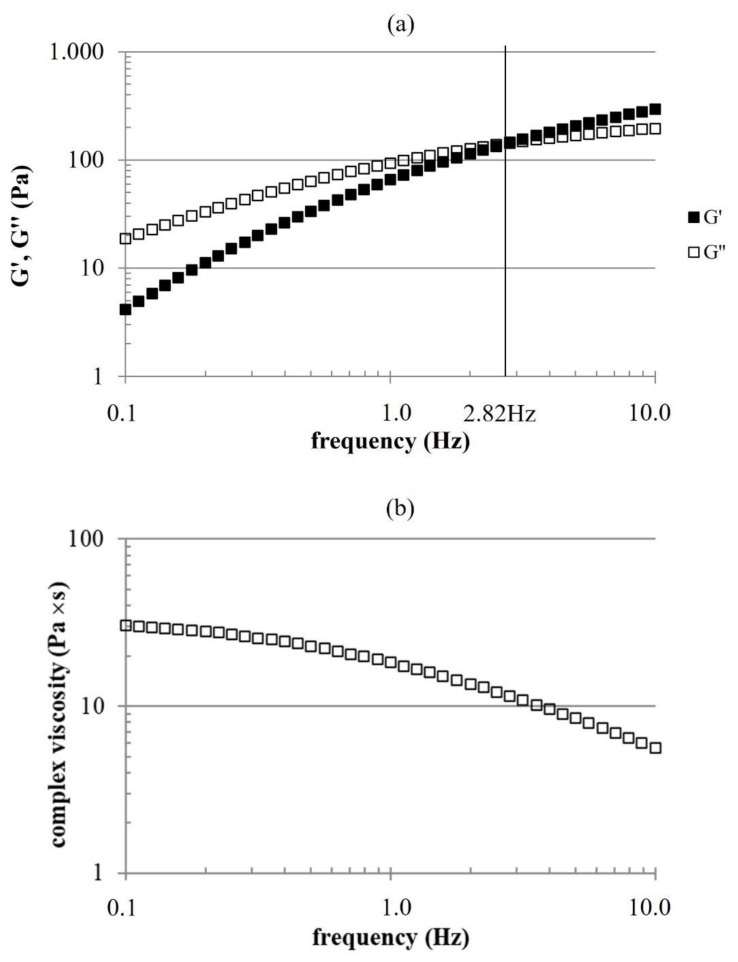
Mechanical spectrum (**a**) and complex viscosity as a function of the oscillation frequency (**b**), recorded at 37 °C, 1% strain.

Amplitude sweep tests revealed a fluid behavior for the HHA/BC sample, with 92 ± 3 Pa and 116 ± 5 Pa G′ and G″ values, respectively. Tanδ in the linear viscoelastic range (LVR) resulted equal to 1.27 ± 0.07. The mechanical spectrum (Figure 1) confirmed fluid behavior (G″ values higher than G′) at low frequencies. Both the moduli progressively increased with the oscillation frequency with G′ rising more markedly than G″. A crossover point was recorded at 2.82 Hz. At higher frequencies, the formulation behaved as elastic with G′ exceeding G″. Complex viscosity decreased from about 30 Pa’s to about 6 Pa’s over the frequency range explored. For BC sample (at 16 mg/mL) G′ and G″ in the linear viscoelastic range were 20 ± 4 mPa and 36 ± 0 mPa respectively, with a relative tanδ of 1.9 ± 0.4. The Complex viscosity was constant with frequency and about 3.5 mPa·s.

### 2.2. Enzymatic Degradation

**Figure 2:**
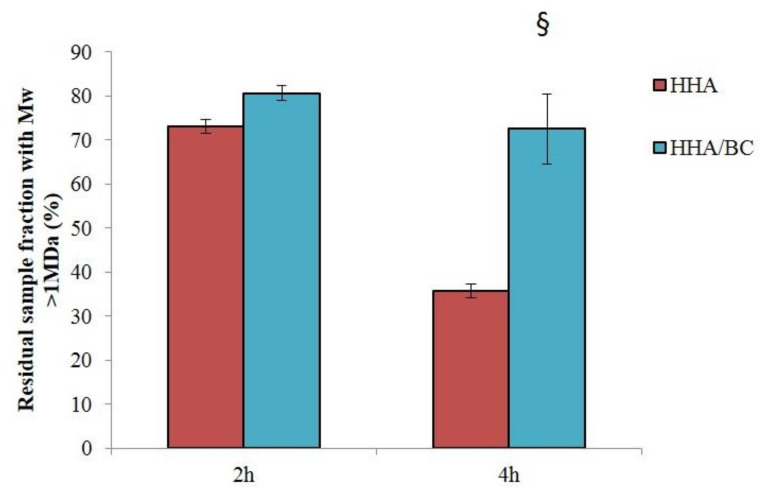
Sensitivity of HHA and HHA/BC preparations to enzymatic hydrolysis. Residual (%) sample fraction with *M*_w_ higher than 1 MDa after 2 h and 4 h of incubation with BTH (1 U/mL) (§ *p* < 0.05 vs. HHA).

HHA/BC stability to enzymatic hydrolysis was tested in comparison to HHA (not combined with BC). Samples proved diverse sensitivity to BTH action (Figure 2). After 2 h of incubation with BTH 1 U/mL, HHA and HHA/BC maintained 73% ± 2 and 81% ± 2 of their high molecular weight fraction (*M*_w_ > 1 MDa), respectively. Differences in stability were more marked at the longest time tested. After 4 h of incubation, HHA/BC still preserved 73% ± 8 of its high molecular weight fraction while 2-fold lower values were recorded for HHA. No significant degradation was observed for BC after incubation with BTH under the conditions used for HHA and HHA/BC. After 6 h of incubation with BTH 50 U/mL, the BC fraction (wt%) with molecular weight higher than 30 kDa was reduced by 76%.

### 2.3. Biological Response

#### 2.3.1. In-Vitro Scratch-Tests on Keratinocytes Monolayers in TLVM

**Figure 3:**
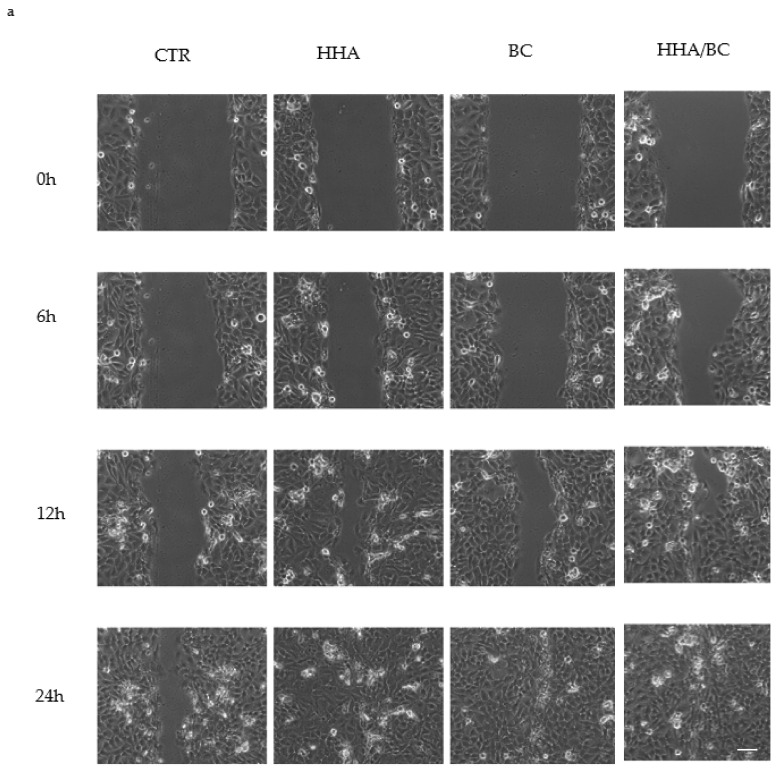

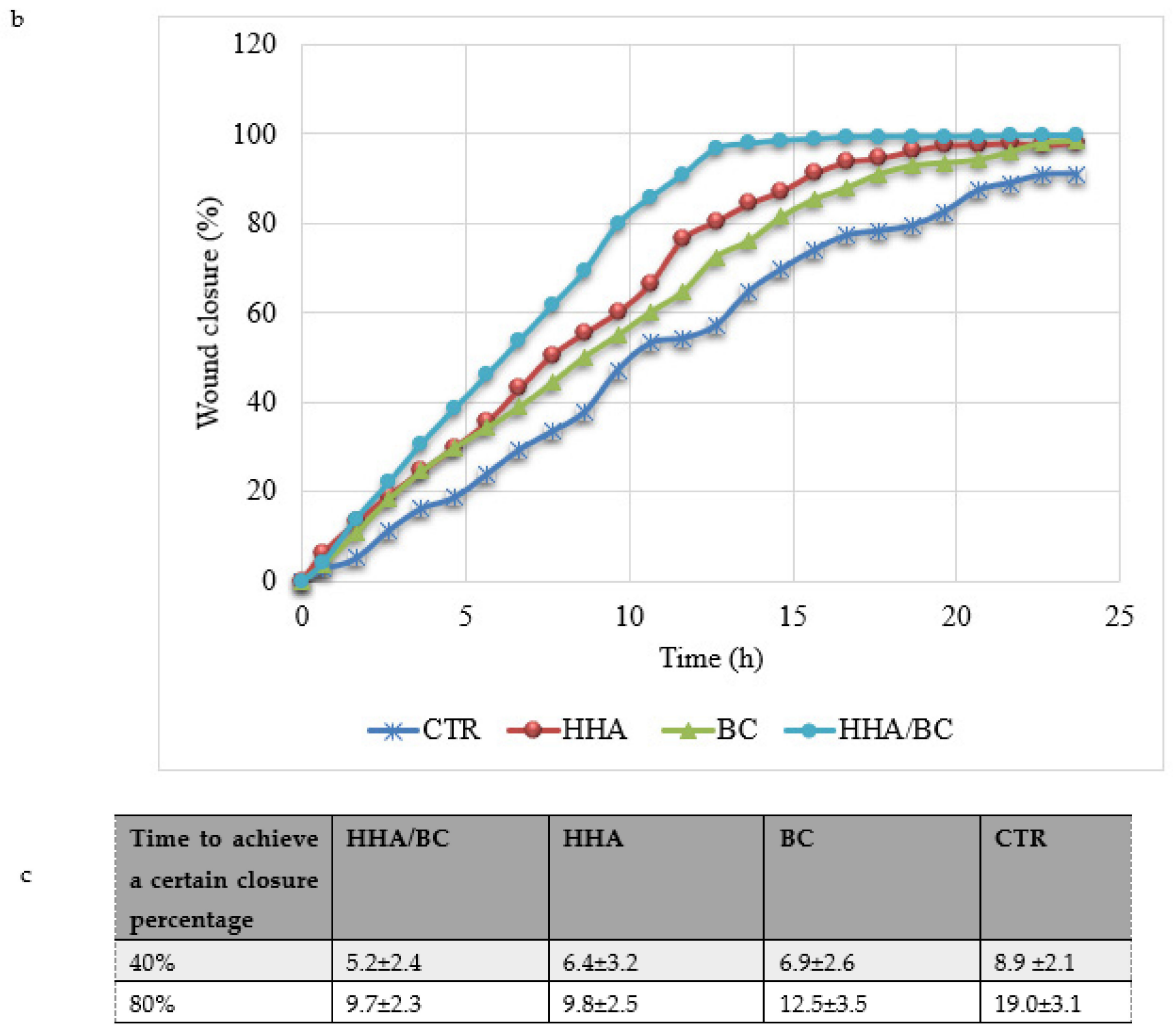
Wound healing experiments/assays. (**a**) Representative fields of view of scratched HaCaT over time in the presence of the different product formulations. CTR, control; BC, biotechnological chondroitin; HHA, high molecular weight hyaluronic acid. Scale bar, 100 μm. (**b**) Quantitative analysis of wound closure [(A_0_ − A/A_0_) × 100] vs. the time. (**c**) Average time (±SD) to achieve a specific percentage of wound closure, namely 40 and 80% for untreated samples (CTR) and in presence of the products.

The results of wound healing experiments are reported in Figure 3.

In particular, micrographs of wound closure (Figure 3a) indicate that HHA/BC prompted faster wound reparation than the sole HHA or BC. The average time needed to reach 40 and 80% wound repair in the presence of the different treatments is reported, showing that the presence of HHA/BC increased the reparation rate by about 2-fold as compared to the control (CTR). A lower improvement of the closure rate was found by treating the cells with HHA and BC alone, highlighting the synergic effect obtained by coupling the two products (HHA/BC).

#### 2.3.2. ECM Gene and Protein Expression in HaCaT after Treatments

**Figure 4:**
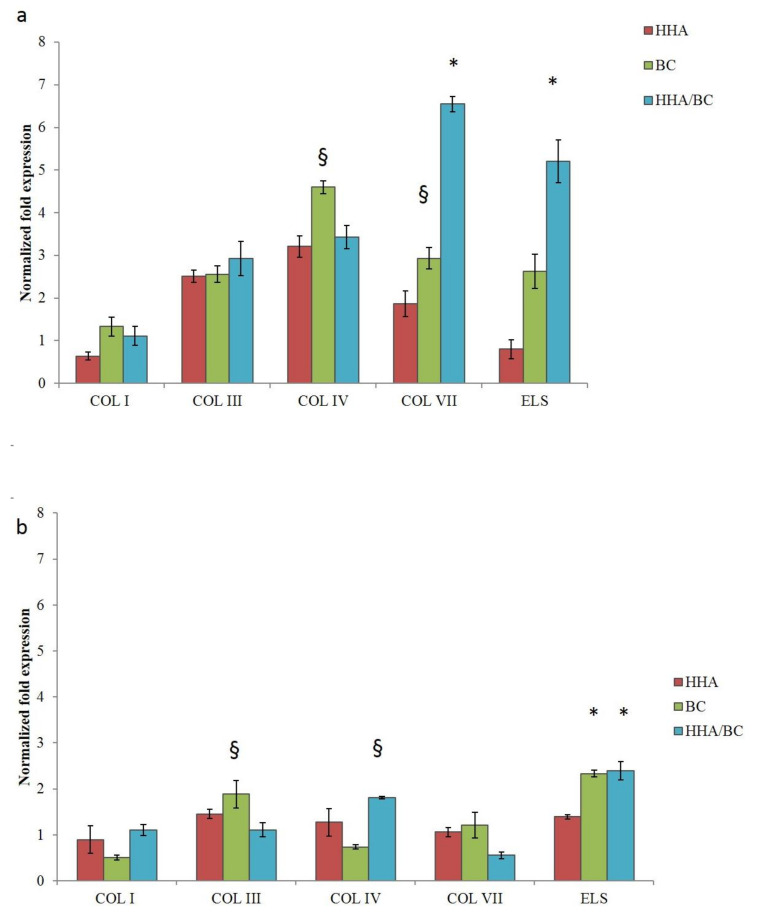
RNA was extracted from HaCat cells. and qRT-PCR were performed to determine the gene expression of *COLI, COL III, COLIV, COL VII* and *ELS* at 4 h (**a**) and (**b**) 24 h. * *p* < 0.01; § *p* < 0.05 vs. CTR. Data are presented as mean ± SD.

In keratinocytes 2D cultures after 4 h incubation (Figure 4a) Type I collagen (*COLI*), type III collagen (*COLIII*), type IV collagen (*COLIV*), type VII collagen (*COLVII*) and Elastin (*ELS*) was investigated. In particular, the expression increase of *COLI* was similar for treatments with BC and HHA/BC and increased compared to that with HHA. The different formulations did not affect the expression of the *COLIII* biomarker (4 h). Expression of *COLIV* was significantly different in samples treated with BC, while supplementation of both HHA e HHA/BC showed similar outcomes. A higher concentration of *COLVII* was found in samples treated with HHA/BC compared to those in which HHA or BC alone were added. Similar results were found for elastin. Compared to HA, BC more efficiently prompted the expression of *COLVII* and elastin. After 24 h, ELS was significantly increased especially in the presence of BC and HHA/BC. BC treatments at 24 h were found to mostly up regulate *COL III* and *COLVII* (Figure 4b).

#### 2.3.3. Dehydration Test

**Figure 5:**
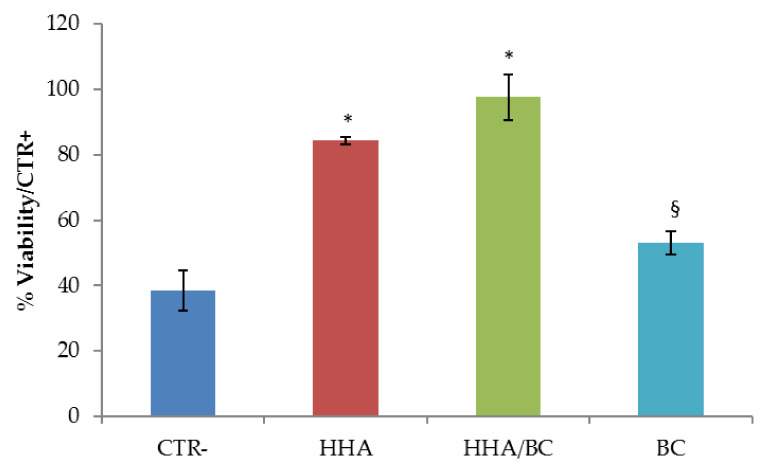
Cell viability of HDF-hTERT exposed to desiccation under no protective conditions (CTR−) and after the potentially protective pre-treatment with HHA/BC, BC and HHA. Cell viability was reported as % in respect to unstressed cells (CTR+). * *p* < 0.01; § *p* < 0.05 vs. CTR.

The potential protective activity of the HHA/BC, BC and HHA samples against dehydration is reported in Figure 5. Viability of cells exposed to desiccation under no protective conditions (CTR−) and of the same treated with HHA/BC and HHA prior to desiccation is reported. Viability was expressed as % in respect to unstressed cells (CTR+). The applied stress was responsible for about 50% mortality (CTR−). Clearly, the pretreatment with HHA/CB, and also with HHA only, resulted in considerable protection to the dehydration. A minor effect of BC in counteracting dehydration was shown but, however, it is still a significant improvement with respect to the control.

Specifically, when exposed to desiccation after being treated with the tested samples, cells preserved viability at about 90%, regardless of the specific preparation used.

#### 2.3.4. 3D FT Skin: ECM Biomarker at Gene and Protein Level and Histological Results

**Figure 6:**
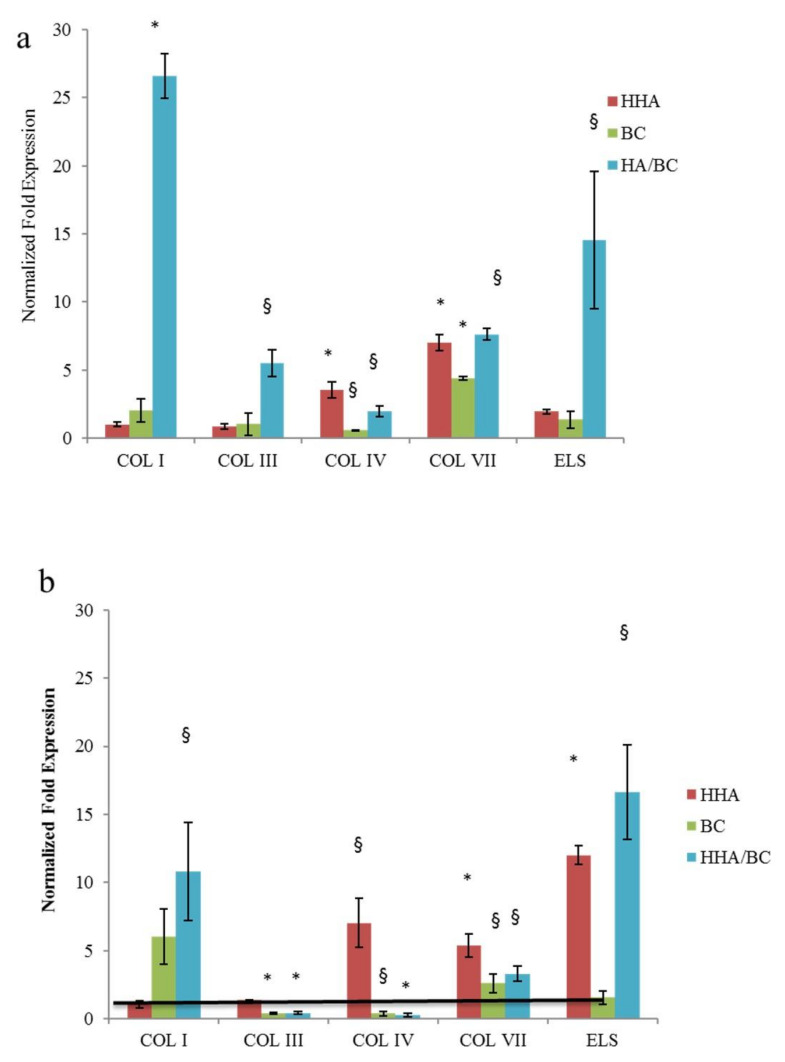
Effect of HHA, BC and HHA/BC on 3D FT-Skin. Data are presented as mean ± SD. At 24 h and 7 days, total RNA was extracted, and qRT-PCR was performed to determine the expression levels of the genes coding for COLI, COL III, COLIV, COL VII and *ELS* at 24 h (**a**,**b**) 7 days. * *p* < 0.01; § *p* < 0.05 vs. CTR.

**Figure 7:**
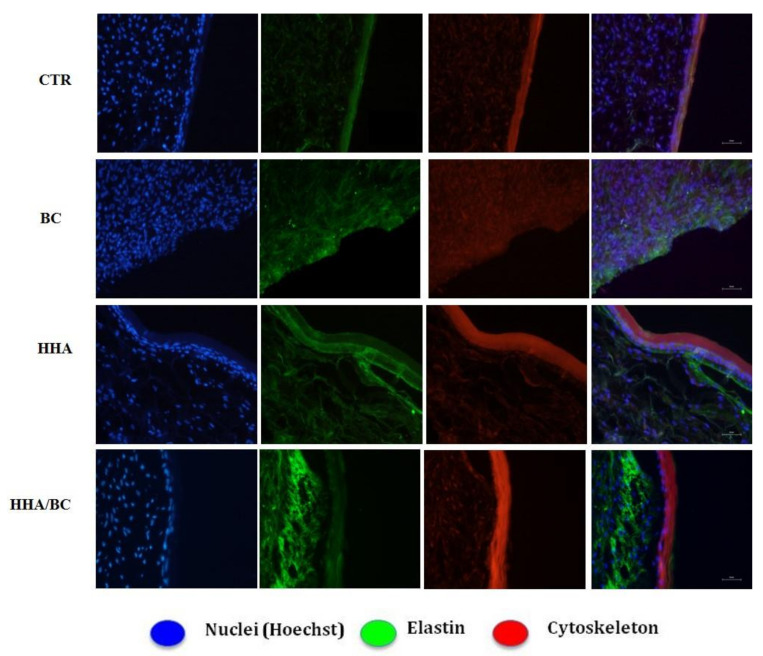
Immunofluorescence elastin staining 7 days after the treatments on 3D skin. Higher elastin expression in the presence of HHA/BC treatments, mainly localized at dermal layer respect to the CTR and HHA and BC alone. Scale bar 100 µm.

In the experiments run on a 3D Full thickness (FT) skin model (Figure 6) the strongest effects are clearly seen in samples treated with HHA/BC which increased *COLI* and *ELS* after 24 h as well as after 7 days. Compared to the others this formulation also increased significantly *ColIII* expression at 24 h. The injection of HHA only resulted in higher levels of *COLIV* and *COLVII* also at both time points whereas ELS seems to be-more expressed only after 7 days. Concerning protein levels, immunofluorescence assays indicated a higher expression of elastin (Figure 7) in the connective tissue (fibroblast zone) when the 3D skin was treated with HHA/BC for 7 days. This highlights an increased elastin content compared to the control and to HA and BC alone.

**Figure 8:**
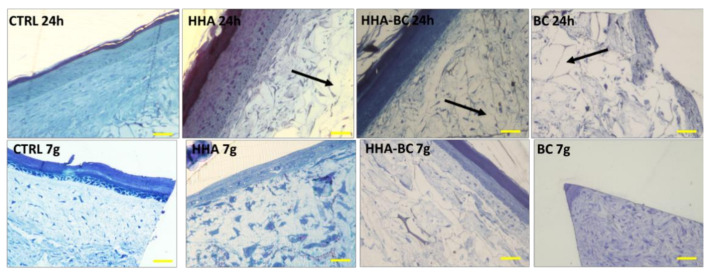
Histological cross section of the full thickness human skin model. Black arrow indicates the injected materials. Mag 20×; scale bar 100 µm.

Histological results (Figure 8) indicated that injections of HHA, BC and HHA/BC did not modify the structure of the FT-skin 3D model, that remained similar to that of healthy human skin. HHA/BC caused an increase of the thickness of the outer layer of the 3D-skin model compared to the control and to the treatment with HHA after 7 days of incubation.

#### 2.3.5. Effect of HHA/BC in an In-Vitro Inflammation Model: HDF + TNFα

- ECM biomarker: gene and protein expression

**Figure 9:**
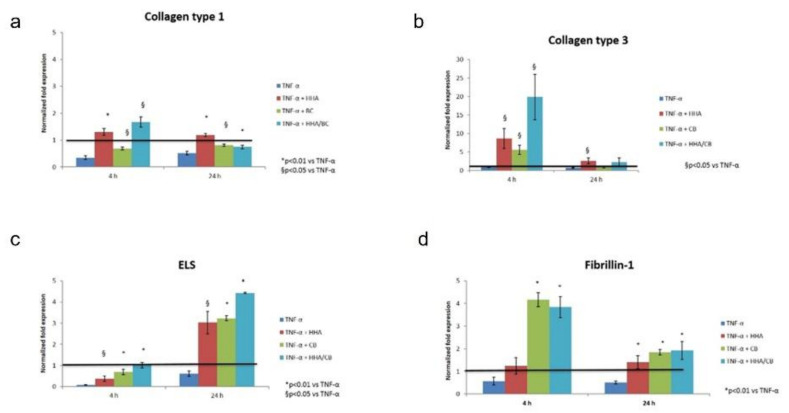
Effect of HHA, BC and HHA/BC on HDF insulted in vitro with TNFα. Data are presented as mean ± SD. qRT-PCR was performed to determine the gene expression of (**a**) Col I, (**b**) Col III, (**c**) ELS and (**d**) FIB-1 after 4 h and 24 h of incubation. * *p* < 0.01; § *p* < 0.05 vs. CTR.

Fibroblasts were challenged with TNF-α, and the expression of ECM biomarkers was monitored following the addition of the three formulations. An upregulation of *COL I* and *III* in the presence of HHA and HHA/BC at early stage (4 h) was found, while elastin expression seemed to be activated later (24 h), and to be higher in the presence of HHA/BC treatments. BC alone significantly affects the expression of *COLIII, ELS* and *Fibrillin-1*. (Figure 9a–d).

- Quantification of Fibrillin (FBN), type I collagen (COL I) and elastin (ELS) protein expression using western blotting

As shown in Figure 9, the TNF-α insult slightly but not significantly reduced *ELS* expression, while *COLI* and *FBN* were almost not affected. As expected, also in this inflammation model, the treatment with hydrogels induced a higher expression of the matrix proteins tested. Specifically, *FBN* concentration was increased by 1.2-fold from H-HA, by 1.3-fold from BC and by 1.7-fold from the HA/BC complex, respectively, compared to the CTR. *COLI* was similar or slightly down-regulated compared to CTR when cells were exposed to HA or BC alone, after addition of TNF- α whereas treatments with the HA/BC complex increased its expression by 1.6-fold vs. CTR.

**Figure 10:**
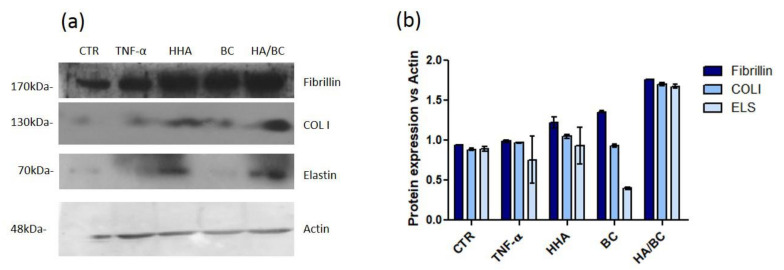
(**a**) Fibrillin, type I collagen and elastin expression determined by western blotting analyses in HDF treated with TNF-α and HHA, BC and HHA/BC samples. (**b**) The expression of each protein was normalized in respect to actin used as housekeeping internal control. Data are reported as average ± SD (*n* = 2).

ELS expression was slightly down regulated in the presence of TNF-α and TNF-α +BC, while H-HA addition after TNF-α insult did not significantly increase *ELS.* Interestingly, HA/BC, added on insulted cells induced a 1.6-fold increase of all protein levels vs. CTR and TNF-α treated cells (Figure 10).

#### 2.3.6. Secreted IL-6 Quantification

**Figure 11:**
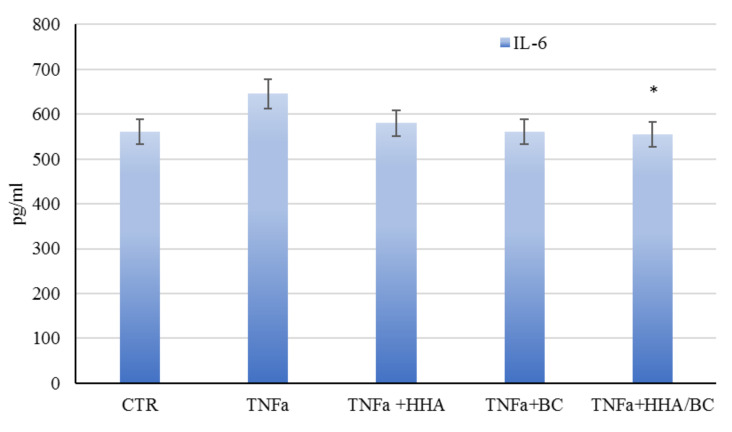
HFD cells treated with TNFα and then treated with HHA, BC and HA/BC samples. Protein expression of cytokines IL-6 by ELISA assay. Samples are in triplicates. * *p* < 0.05 vs. TNFα.

The results reported in Figure 11 showed a slight reduction of IL-6 in the presence of HHA/BC formulations and of BC alone.

#### 2.3.7. Melanin Content in HaCaT/HEMa-LP Co-Cultures

**Figure 12:**
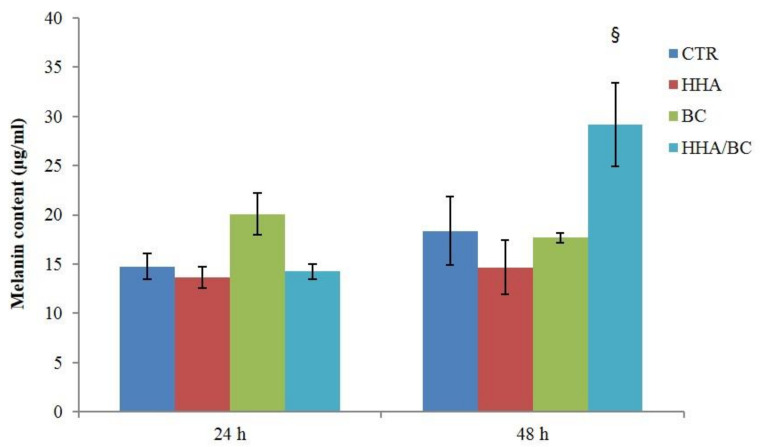
Melanin content in lysed HaCat/HEMa-LP co-colture after 24 and 48 h of HHA, BC and HHA/BC treatments. § *p* < 0.05 vs. CTR.

The content of melanin was evaluated in cell extracts recovered from co-cultures of HaCaT and HEMa-LP, as described in the Section 4 (Materials and Methods). Results after 24 and 48 h of incubation with all formulations were reported in Figure 12. Data showed that after 24 h, only BC slightly raised melanin content, whereas prolonging the incubation (48 h), HHA/BC significantly increased melanin concentration by 1.6-fold compared to CTR.

## 3. Discussion

The function of HA in tissue hydration is well established and its activity in promoting the biosynthesis of matrix constituents has also been demonstrated in vitro. However, better understanding of the diverse bioactivities during the multiple stages of tissue regeneration, such as the antioxidant and/or anti-infective action (i.e., in wounds) and the modulation of inflammation markers, is still under investigation. Recently, (hybrid) cooperative complexes made of high and low molecular weight HA were designed, studied, and produced as injectable medical devices and the in vitro outcomes helped clinicians to make proper use of these products.

The formulation here described based on HHA/BC represents a further opportunity of innovation due to the availability of biofermentative unsulfated chondroitin. In fact, up to date all chondroitin-based formulations distributed on the market exploit extractive sulfated chondroitin (e.g., bovine trachea or shark fins, or other specialized tissues in swine). Biotechnological processes overcome safety issues and also respond to a growing awareness for patients that may have ethical or religious concerns, or simply consider the extractive product less safe. Nevertheless, CS has been studied and used in medical devices and biomaterials/3D scaffolds. For instance, it is a functional component in a dermal substitute [7] used to treat diabetic foot ulcers. A recent study suggested that the bioactivity of chondroitin sulfate in wound healing is correlated to the sulfation pattern, but not to the source from which the material was extracted. These results were achieved by comparing extractive and semisynthetic CS, with diverse sulfation patterns [14]. BC is a galactosamine-glycan of about 35 kDa (like shark derived CS) and, due to its composition and to the absence of sulfate groups, it shows similarity to both CS and HA, respectively. Although charge density seems to have a crucial role in cell-based interactions, even BC alone demonstrated to induce wound repair in scratch assays performed in this study. Moreover, supplementation of BC by itself also resulted in the increase of ECM components’ biosynthesis showing its suitability for dermal applications. The potential of this molecule was further investigated by combining it with HHA. The characterization of the resulting formulation revealed the possibility to (a) increase the delivered amount of GAGs, due to the formation of cooperative hydrogen bonds (namely, hybrid complexes) that reduce viscosity and improve injectability; (b) enhance the storage modulus whilst preserving flowability to customize the properties of injectable formulations; (c) slow down product degradation, since the complexes can, to a certain extent, shield HHA from the attack of BTH; (d) potentially improve extracellular matrix remodeling, as a result of a two-components synergic effect. These points will be discussed in the next paragraphs.

Intradermal injectable preparations should satisfy certain requirements in terms of flow properties. Specifically, injectability as well as proper mechanical behavior after delivery are required. The rheological profile obtained for the novel HHA/BC supports injectability. The preparation was easily extruded even with fine bore needles (e.g., 29–30 gauge) (data not shown). This is noteworthy since HHA/BC is the most concentrated formula for intradermal delivery proposed so far compared to the average in commercial formulations. It would allow delivery of up to 40 mg/mL of GAGs and might make it feasible to even further increase the concentration of hybrid cooperative complexes by 25% (e.g., PROFHILO^®^ 32 mg/mL).

The mechanical spectrum highlighted viscoelastic properties typical of entangled networks with relative dynamic moduli values indicating a rheological behavior turning from viscous to elastic with increasing frequency, similarly to properties of HHA solutions [16]. It is interesting that, due to the position of G′-G″ crossover, the formula behaves similarly to crosslinked products over a rather wide range of frequencies [17,18]. This supports a good biomechanical response, once intradermally delivered, even in the absence of chemical modifications of the biopolymers. BC, because of the low *M*_w_, presented much lower moduli, as expected.

Data on the sample’s sensitivity to enzymatically catalyzed hydrolysis are key since depolymerization affects the formula’s rheological behavior. The improved resistance to BTH hydrolysis recorded for HHA/BC compared to HHA is in line with previous studies on similar formulations combining HHA and low molecular weight HA (LHA) [2]. BC proved sensitive to BTH as expected considering BC structural similarity to HA. Sensitivity was far lower compared to HHA (BC required 50-fold higher enzyme concentration to significantly reduce the sample *M*_w_) consistently with the lower biopolymer size [19].

Tissue regeneration and remodeling were also investigated. In particular, scratch-tests on human cell monolayers in time lapse experiments proved a higher regenerative effect of HHA/BC compared to HHA and BC alone, as previously demonstrated for HA based complexes [2]. The remodeling properties of the products were established by assessing their capacity to re-activate the turnover of ECM components (type I, III, IV, VII collagen and elastin), which is fundamental for skin regeneration processes. In particular, type I and III collagen are the most abundant types of collagens in skin (about 80–85% and 10–15%, respectively) [20] and play central roles during collagen fibers formation. Type III collagen is more prevalent in young skin than aged skin and is particularly involved in wound healing [21,22].

However, the alteration of biosynthesis rate and the modification of collagens relative abundance is known to occur as a consequence of aged or injured skin tissue [23]. Collagen type IV is the primary collagen found in basal lamina enabling keratinocyte and fibroblast migration and adhesion to the basement membrane and is characterized by a more flexible and kinked conformation [24]; instead, anchoring fibrils are constituted of type VII collagen whose basal expression was shown to decrease in an age-dependent manner in fibroblasts [25]. Collagen VII has been shown to support dermal fibroblast migration and, additionally, modulates cytokine expression in wound-infiltrating inflammatory cells. Several studies proved the importance of COLIV and VII in the wound healing process [26]. In this respect, it is demonstrated that alterations and mutations of the genes of the type IV and VII collagens are found in pathological skin condition [27,28]. Given the importance of all these different types of collagens, we included in this study also the COL IV and VII, already analyzed in our previous manuscript [2]. Apart from the alteration of collagen fibers, in aged skin, elastic fibers present significant signs of elastolysis. For this reason, notwithstanding the minor representativity of elastin in the dermis (accounting for 2–4% of ECM), an enhancement of its biosynthesis represents a target for an ideal dermal injectable gel [29]. Comparative analysis of H-HA/BC treatments in respect to H-HA and BC in linear form and to untreated cells (control) were carried out, and the gene expression levels of collagens (as referred above) and elastin were evaluated by quantitative real-time PCR (RT-qPCR). Specifically, in 2D cultures, COL I was not modulated at either of the investigated time points confirming its lower expression on the superficial epidermal layer in respect to connective tissue [2]. Interestingly, the increase of COL IV, VII and ELS at the transcriptional level in the presence of HHA/BC was observed. Moreover, the un-sulfated chondroitin, for the first time assayed on dermal cells, increased collagens expression (e.g., *COLIII, IV* and *VII*) and elastin up to 24 h of treatment. As reported by Nakab and collaborators, the density of dermal collagen could be an indicator of skin integrity, since collagen’s function is to sustain skin strength, helping it to withstand mechanical deformation [30]. Therefore, it seems that besides HA, also BC and combined formulations may contribute to tissue reparation.

Elastin was also quantified using the 3D Skin Model to better resemble natural dermis in vivo. As previously demonstrated, HHA stimulates the production of collagen and elastin, in different cell models and also in 3D [2]. In this study these results were confirmed, showing that the presence of BC in the HHA-BC complex even amplifies the effect of HHA alone. These data may be due to the persistence of the GAGs at injection site, but also possibly to the concurrent biochemical interaction of HHA and BC gradually released from the entangled matrix obtained by a patented thermal process. (NaHyCo technology)

Finally, the novel formulation (HHA/BC) was evaluated in an inflammation model, specifically aiming at resembling stretch marks. The latter can be described as scars with microscopic evidence of flattening of the epidermis and consequent reduction of melanocyte cells (HEMa-LP), that also present thinning and retraction of the dermal collagen and elastin [31]. Since this epidermal degeneration is linked to ECM damage/modification and inflammation, the in-vitro approaches consisted in the evaluation of HHA/BC hydrogels as anti-inflammatory agents on TNF-α treated fibroblasts [32]. As demonstrated by the increased expression of COLI, III, ELS and Fibrili-1 in TNF-α insulted cells, HHA/BC affects matrix regeneration, counteracting inflammation. Finally, HHA/BC formulations proved to modulate melanogenesis, by increasing melanin biosynthesis, an alteration that occurs in the presence of scar tissue such as in stretch marks.

## 4. Materials and Methods

### 4.1. Materials

Biofermentative chondroitin (BC) was obtained in our laboratory. An extended purification protocol based on ultrafiltration/diafiltration, activated carbon treatments and ethanol precipitation was accomplished to obtain a purity of about 95 ± 5% *w*/*w* [33]. In addition, endotoxin concentration of BC containing gels (EU/mL) was evaluated using the Limulus test; samples with a titer lower than 0.25 EU/mL were considered suitable for further applications.

Highly purified high-molecular weight hyaluronic acid (H-HA; *M*_w_ 1200 ± 100 kDa) was produced by Altergon s.r.l., Morra De Sanctis, Avellino, Italy, from proprietary strain *Streptococcus equi* as a fermentative ultrapure pharmaceutical grade HA (i.e., purity > 95%, water content < 10%, EU/mg < 0.05, extremely low heavy metals content).

DMEM, FBS; Trypsin, were purchased by M&M Biotech (Naples, Italy)

All solutions were prepared dissolving gels of HHA, BC (at 32 mg/mL) and their combination (24 mg/mL HHA and 16 mg/mL BC) in phosphate buffer; successively they were thermally treated according to the patented protocols [34]. Resultant gels were then characterized from a biophysical point of view and used for biological assays. To this aim, the formulations were diluted in the medium (DMEM, added with 1% FBS) at a final concentration of 5 mg/mL (0.5% *w*/*w*).

After dilution, solutions were microfiltred (0.22 µm) before use in cell-based experiments to further reduce potential contamination issues/risks.

### 4.2. Rheological Studies

Measurements were carried out using a Physica MCR301 oscillatory rheometer (*Anton Paar*, Ostfildern, Germany) as previously reported with slight modifications [16,18]. Amplitude sweep and frequency sweep tests were performed at 37 °C, using a CP 50-1 geometry (cone diameter 49.968 mm, cone angle 0.994°, truncation 100 µm). Amplitude sweep tests were carried out at 1.59 Hz frequency, over a strain amplitude range of 0.1–100%. The linear viscoelastic range (LVR) was derived as the range of amplitude over which constant values for the moduli were recorded. Oscillation frequency sweep tests were then carried out over a frequency range of 0.1–10 Hz at a constant 1% strain (within the LVR). Complex viscosity as a function of the frequency was also derived. For the BC sample, a h a Double Gap geometry (DG26.7Q1-SN42960) with a measuring gap (internal) of 0.419 mm was used.

### 4.3. Stability to Enzymatic Hydrolysis

Formulation sensitivity to enzymatic degradation was studied as previously reported, with slight modifications [17,35]. Bovine testicular hyaluronidase, BTH (EC 3.2.1.35), salt-free lyophilized powder with a specific activity of 1275 U/mg was purchased from Sigma-Aldrich S.R.L. (Milan, Italy). Samples were diluted (in phosphate buffer, pH 7.4) to 1% *w/v* and were incubated in the presence of BTH (1 U/mL), at 37 °C, under stirring (1000 rpm). At increasing incubation times up to 4 h, the samples were withdrawn, boiled for 10 min to inactivate the enzyme, and then centrifuged (5 min, 13,000× *g*). The sample was recovered, filtered using 0.22 μm disposable filters and appropriately diluted for the following analyses. Samples were then characterized using a Size Exclusion Chromatography-Triple Detector Array (SEC-TDA) equipment by Viscotek (TDA305), Malvern, UK). The SEC-TDA technique allowed for a comprehensive hydrodynamic characterization of the polymeric samples; in particular, the weight average molar mass (*M*_w_), the numeric average molar mass (Mn), the polydispersity index (*M*_w_/Mn), the intrinsic viscosity ([η]) and the hydrodynamic radius (Rh) were derived. The sample fraction (wt%) exhibiting *M*_w_ higher than 1 MDa was also derived. Stability was evaluated by monitoring the variation of the high molecular weight fraction in the sample over incubation with the enzyme. BC degradation in the presence of BTH was evaluated by monitoring the maintenance (%) of the sample fraction (wt%) with molecular weight higher than 30 kDa.

**Figure 13:**
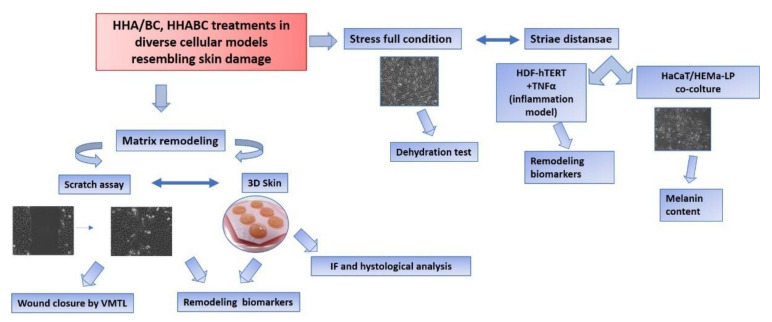
Schematic diagram that shows the experimental set up.

An overview of biological experiments was reported in Figure 13.

### 4.4. In-Vitro 2D- and 3D-Skin Models

A spontaneously transformed non-tumorigenic human keratinocyte cell line (HaCaT) (purchased from Istituto Zooprofilattico, Brescia, Italy) was cultured in Dulbecco’s Modified Eagle Medium (DMEM) supplemented with 10% (*v*/*v*) heat inactivated fetal bovine serum (FBS), 100 U/mL penicillin and 100 μg/mL streptomycin. All materials were purchased from Gibco (Thermo Fisher scientific, Monza, Italy). The cells were grown on tissue culture plates (Corning Incorporated, New York, NY, USA), using an incubator with a humidified atmosphere (95% air/5% CO_2_ *v*/*v*) at 37 °C. Collagen type 1, for coating, was purchased from Sigma Aldrich (Milan, Italy). HaCaT monolayers were used for wound healing experiments and to evaluate gene expression and protein levels of specific biomarkers.

Human Epidermal Melanocytes (HEMa-LP, Gibco, ThermoFisher Scientific, cod. C0245C) were isolated from lightly pigmented (LP) adult skin. The cells were cultured in M-254 medium (cod. M254500) supplemented with HMGS-2 (cod. S0165), penicillin (100 U/mL), gentamicin (10 mg/mL) and amphotericin B (0.25 mg/mL) at 37 °C in 5% CO_2_. HEMa-LP were used in co-culture with HaCaT for the evaluation of melanin content.

A human dermal fibroblasts cell line immortalized with hTERT (HDF cells, BJ-5ta, ATCC CRL-4001), was cultured in DMEM/F12 (Gibco) supplemented with 10% FBS (*v*/*v*).

The Phenion^®^ Full Thickness Skin Model, produced by Henkel (Düsseldorf, Germany, diameter 1.3 cm) was used for the 3D-experiments. It consists of epidermal keratinocytes and dermal fibroblasts (obtained from biopsy material of healthy donors) organized in a multilayer that resembles human skin tissue structure and functionality [36].

The experiments were run following a previously described protocol [2]. Briefly, Hybrid complexes (HHA/BC) and HHA or BC gels were injected in six different points (50 μL each) of the FT-SKIN specimen just below the epidermal surface. The samples were then incubated for 24 h and 7 days. Control samples were injected with PBS solution (6 injections of 50 μL. Type I collagen (COLI), type III collagen (COLIII), type IV collagen (COLIV), type VII collagen (COLVII) and Elastin (ELS) were evaluated at the transcriptional and protein level. Moreover, supernatants at 24 h, and 7 days were collected and stored at −20 °C in order to test HBD-2 release, by using an ELISA assay.

All materials for HDF cultures were purchased from LGC Standards S.r.L., Sesto San Giovanni, (Milan, Italy). The cells were grown on tissue culture plates (BD Falcon, Naples Italy), using an incubator with a humidified atmosphere (95% air/5% CO_2_ *v*/*v*) at 37 °C. HDF were also extracted at the end of the experiments to quantify gene and protein expression for selected biomarkers. HDF cells were treated with TNF-α 10 ng/mL, in order to mimic an in vitro inflammation condition for the connective tissue [32,37], and incubated for 24 h following addition of the three diverse formulations.

### 4.5. In-Vitro Scratch-Tests on Keratinocytes Monolayers in Time Lapse Experiments (TLVM)

The effects of the different HHA/BC formulations were studied using the well-established scratch-test in time lapse experiments. The model was used in diverse research projects in our lab and described in recent literature reports [38]. Briefly, 1.5 × 10^5^ HaCaT cell monolayers were mechanically scratched using a sterile pipette tip (Ø = 0.1 mm) taking care to create uniformly sized wounds of approximately 0.5–0.9 mm in width. All cell debris were removed by washing twice with a PBS solution. Scratched monolayers were then treated with different solutions containing HHA and BC alone and in combination (HHA/BC 1.5:1) at a concentration of 0.5% *w*/*w*. The cell culture medium, DMEM supplemented with 1% FBS, was used as a control. The multiwell was then accommodated in the CO_2_ microscope stage incubator of the time lapse station where all the required environmental conditions for cell cultures are maintained. Observation and quantitative analyses of cell migration were performed by time lapse video-microscopy for 48 h, as fully reported elsewhere [38].

### 4.6. RNA Isolation and Quantitative Gene Expression by Real-Time PCR (qRT-PCR) on HaCaT, HDF Cells and FT-Skin Model

Total RNA was extracted from control and treated HaCaT, HDF monolayers and FT-Skin model at different time points (4 and 24 h for 2D culture and 24 and 7 days for FT-Skin) using TRIzol^®^ (Invitrogen, Milan, Italy), according to the manufacturer’s procedures described in literature [39,40]. Briefly, extracted RNA was quantified with a Nanodrop spectrophotometer (Celbio, Milan, Italy) and 1 μg of DNase-digested total RNA was used (DNA-free kit; Ambion-Applied Biosystems) to synthesize cDNA using the Reverse Transcription System Kit (Promega, Milan, Italy). Quantitative RT-PCR was performed with the iQ™ SYBR^®^ Green Supermix (Bio-Rad Laboratories s.r.l., Milan, Italy) to evaluate dermal remodeling-specific markers (type I, III, IV and VII collagens, elastin and fibrillin-1) after 4 and 24 h of incubation. The primer sequences were designed by Beacon DesignerTM software (Bio-Rad Laboratories S.r.l. V, Segrate (Milan, Italy) and are shown in Table 1. All the samples were assayed in triplicate, and the hypoxanthine guanine phosphoribosyl transferase (HPRT) housekeeping gene was used to normalize the mRNA expression of analyzed genes. The variations were calculated using the comparative threshold method (∆∆Ct = difference in ∆Ct between GAG-treated cells and control), and the results are reported as the normalized fold expression using the quantification of 2^−ΔΔCt^ method through Bio-Rad iQ5 software (Bio-Rad Laboratories, Milan, Italy).

### 4.7. Dehydration Test

The dehydration test was performed according to previous reports with modifications [41]. HDF-hTERT were used as cellular model. Cells were seeded in a standard 24-well culture plate, until ~50% confluence for optimal observation of morphological features. At this point, culture medium was replaced with fresh culture medium containing HHA/BC, HHA and BC. For the negative controls CTR−) de-hydration occurred without GAGs addition. After 2 h incubation under cell culture conditions, GAGs-treated cells and not-treated cells (CTR−) underwent de-hydration: medium was removed and cells were left dehydrating (~30 min), until morphological changes indicating a response to the applied stress, was observed. Untreated cells not undergoing dehydration were used as positive control (CTR+). An MTT(3-(4,5-dimethylthiazol-2-yl)-2,5-diphenyltetrazolium bromide) test was then carried out to measure cell viability for formula-treated cells, CTR+ and CTR− cells. The experiment was performed in triplicate. Cell viability was reported as percentage (%) (mean value ± SD) on viable cells in the control sample.

### 4.8. Fibrillin (FBN), Type I Collagen (COL I) and Elastin (ELS) Protein Determination by Western Blotting

Western blotting analyses were performed to analyze Fibrillin (FBN), type I collagen (COL I) and Elastin (ELS) protein expression level. In brief, after 24 h HDF untreated-cells and the ones treated with the diverse gels (H-HA/BC, H-HA and BC concentration 0.5% *w*/*v*) were harvested and lysed in RIPA buffer (Cell Signaling Technology, Danvers, MA, USA). Protein concentration was obtained using the Bradford method. Equal amounts of protein (20 µg) were separated by electrophoresis on 10% *w*/*v* SDS-PAGE and transferred to a nitrocellulose membrane (GVS, Life Sciences, North America, Sanford, FL, USA). After blocking with 5% *v/v* nonfat milk in Tris-buffered saline and 0.05% *w/v* Tween-20 (TBST), the membrane was incubated with primary antibodies used at 1:250 dilutions overnight at 4 °C. Immunoreactive bands were detected by chemiluminescence using corresponding horseradish peroxidase-conjugated secondary antibodies (Santacruz Biotechnology, Wembley, UK, 1:5000 dilutions) and reacted with an ECL system (Chemicon-Millipore, Via Monte Rosa 93 20149, (Milan, Italy). Protein levels were normalized with respect to the signal obtained with anti Actin polyclonal antibody (Santacruz Biotechnology, UK 1:500 dilutions). The semi-quantitative analysis of protein levels was carried out by using the Gel Doc 2000 UV System and the Gel Doc EZ Imager, with the quantity one software (Bio-Rad Laboratories S.r.l. Via Cellini, 18/A, Segrate (Milan, Italy).

### 4.9. IL-6 Quantification by ELISA

Supernatants of HDF cell cultures challenged with TNF-α, (CTR−) and eventually added with HHA, BC and HA/BC formulations, were analyzed to quantify IL-6 production using ELISA assay following to the manufacturer’s suggested protocol (Boster antibody and ELISA experts, Tema Ricerca, Padova, Italy). In brief, after 24 h of treatment, cell supernatants were collected and centrifuged (3000 rpm for 10 min at 4 °C) and then transferred to a microtiter plate for incubation with specific biotinylated polyclonal antibody for 1 h. Each experiment was performed in triplicate and optical densities were measured at 450 nm using a microplate reader (Biorad laboratories, S.r.l., Segrate (Milan, Italy). The analytic concentrations were calculated using a standard curve according to the manufacturer’s instructions and as previously reported [42].

### 4.10. Histological Analyses on 3D FT-Skin

FT-skin samples were fixed in 2.5% *v/v* glutaraldehyde in 0.1 M cacodylate buffer (pH 7.4) overnight at 4 °C, de-hydrated in increasing ethanol percentages and embedded in epoxy resin. All products were purchased from Sigma Aldrich (Milan, Italy) Semithin sections (1 μm), obtained through Reichert E ultramicrotome were stained with toluidine blue, observed and photographed at Nikon Eclipse Ci microscope.

### 4.11. Elastin Immunofluorescence Staining on 3D-Skin Sections

Elastin protein levels were detected on paraffin-embedded FT-Skin tissues by immunofluorescence staining as previously described in Stellavato et al., 2016 [2]. Briefly, FT-skin sections, injected and incubated for 7 days with the above-mentioned formulations, were fixed in formalin 4% *v/v* in PBS overnight. Specific dehydration steps with increasing ethanol concentration (until 95% *v*/*v*), followed by isopropyl alcohol, were run on the FT sections. Then, a treatment with histological grade xylene (Sigma, Aldrich, Milan, Italy) overnight was accomplished in order to finally allow paraffin inclusion. Before elastin antibody incubation, slices (10–20 μm thick) obtained by cutting with a microtome were de-paraffinated and rehydrated; 8 min incubation at 99 °C in citrate buffer (used as antigen retrieval) was next carried out. Tissue slices were blocked with 5% BSA in PBS for 60 min and incubated with the primary antibody: elastin (monoclonal mouse antibody, 1:50; Santa Cruz Biotechnology, Dallas, TX, USA). After this, the sections were incubated with the appropriate secondary antibody, Alexa Fluor 488-conjugated secondary antibody (Invitrogen, Milan, Italy) for 1 h. Staining with fluorescent phalloidin TRITC conjugate (Sigma-Aldrich, Milan, Italy) (50 mg/mL in PBS solution) for 40 min at room temperature was used to visualize actin filaments. Nuclei were stained with Hoechst for 10 min (0.5 μg/mL Sigma-Aldrich, Italy). Immunofluorescence images were acquired through accurate examination and analysis by Nikon fluorescence microscope (Multicolor Package, Leica, Milan, Italy).

### 4.12. Melanin Quantification in Keratinocytes/Melanocytes Co-Cultures

A melanin content assay on supernatants recovered from HaCaT keratinocytes/melanocytes (HEMa-LP) co-coltures was performed using a ratio 5:1 of HaCaT/HeMa-LP as previously reported [15,43]. In brief, 7 × 10^4^ HaCaT cells were seeded in each well of 12 well-plates; after 24 h 1.4 × 10^4^ HeMa-LP cells were added to each well containing HaCaT, which were about 30% confluent. Co-cultures of melanocytes and keratinocytes were maintained in DMEM 10% FBS (medium for HaCaT), for 24 h. Successively cells were incubated with the different formulations, opportunely diluted in DMEM 10% *v/v* FBS. The melanin content was evaluated after 24 h and 48 h of treatment. At each time point, cells were washed three times with PBS and lysed with 200 µL 1 N NaOH. For the quantification, the crude cell extracts were transferred into 96-well plates and absorbance at 405 nm (wavelength for melanin maximum absorbance) was measured using a microplate reader (Infinite 200 PRO-Life Sciences-Tecan, Männedorf, Switzerlan). Experiments were performed in triplicate. Synthetic melanin (purchased from Sigma-Aldrich, cod.M8631) was used as standard to build up a calibration curve (0–400 µg/mL) for each experiment to extrapolate the melanin concentration in the diversely treated samples.

## 5. Conclusions

A multilevel in-vitro approach, using diverse cell lines and models, was applied in a study for the first time, with the potential to use biofermentative unsulfated chondroitin in the treatment of damaged skin. Data indicated that BC supports dermal cell viability and migration, prompting wound healing and modulates ECM components by significantly increasing their biosynthesis. Compared to HHA and BC alone, the combined formula (HHA/BC) more efficiently induced remodeling of dermal extracellular matrix and reduced inflammation. The rheological behaviour indicates the suitability of the product for intradermal delivery. All these outcomes suggest the novel formulation as potential new treatment in several applications related to dermatology and aesthetic and regenerative medicine.

## Figures and Tables

**Table 1:** Primer sequences for specific biomarkers employed for the qRT-PCR.

Gene Name (Symbol)	PCR Primer Sequence 5′→ 3′	Amplicon Length (bp)
Ipoxantina-guanina fosforibosiltransferasi (HPRT)	CATCCTGCACCACCAACTG CACAGTCTTCTGAGTGGCAG	117
Type I collagen (COLIA1)	CCAGAAGAACTGGTACATCA CCGCCATACTCGAACTGGAA	103
Type III collagen (COLIIIA1)	TGGTCCCCAAGGTGTCAAAG GGGGGTCCTGGGTTACCATTA	106
Type IV collagen (COLIVA1)	GGATCGGCTACTCTTTTGTGATG AAGCGTTTGCGTAGTAATTGCA	104
Type VII collagen (COLVIIA1)	CGGAACTGACCATCCAGAAT AATAGGGTGCTCACGGTCAC	104
Elastin (ELS)	AGGTGTATACCCAGGTGGCGTGCT CAACCCCTGTCCCTGTTGGGTAAC	105
Fibrillin 1 (FBN-1)	GTCAGATAGCTCCTTCCT TCTGGCATAGACAGTGAT	110

## Data Availability

All data are reported in the manuscript, graphs and table. Row data are available on request to the correspondent author.
